# Hydrophobic Mismatch in the Thylakoid Membrane Regulates Photosynthetic Light Harvesting

**DOI:** 10.1021/jacs.4c05220

**Published:** 2024-05-17

**Authors:** Sam Wilson, Charlea D. Clarke, M. Alejandra Carbajal, Roberto Buccafusca, Roland A. Fleck, Vangelis Daskalakis, Alexander V. Ruban

**Affiliations:** † Department of Biochemistry, School of Biological and Behavioural Sciences, Queen Mary University of London, London E1 4NS, United Kingdom; ‡ Centre for Ultrastructural Imaging, King’s College London, London SE1 1UL, United Kingdom; § Department of Chemistry, School of Physical and Chemical Sciences, Queen Mary University of London, London E1 4NS, United Kingdom; ∥ Department of Chemical Engineering, School of Engineering, University of Patras, Patras 26504, Greece

## Abstract

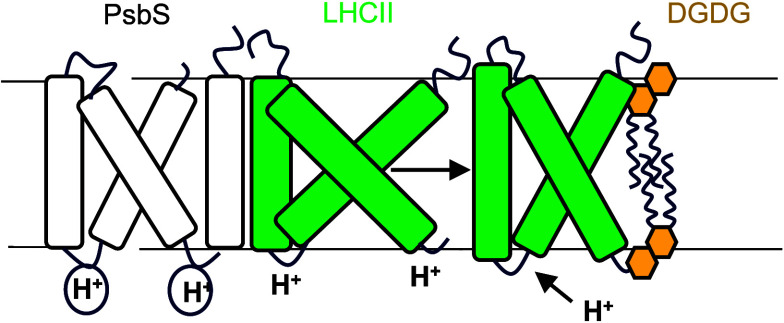

The ability to harvest light effectively in a changing environment is necessary to ensure efficient photosynthesis and crop growth. One mechanism, known as qE, protects photosystem II (PSII) and regulates electron transfer through the harmless dissipation of excess absorbed photons as heat. This process involves reversible clustering of the major light-harvesting complexes of PSII (LHCII) in the thylakoid membrane and relies upon the ΔpH gradient and the allosteric modulator protein PsbS. To date, the exact role of PsbS in the qE mechanism has remained elusive. Here, we show that PsbS induces hydrophobic mismatch in the thylakoid membrane through dynamic rearrangement of lipids around LHCII leading to observed membrane thinning. We found that upon illumination, the thylakoid membrane reversibly shrinks from around 4.3 to 3.2 nm, without PsbS, this response is eliminated. Furthermore, we show that the lipid digalactosyldiacylglycerol (DGDG) is repelled from the LHCII-PsbS complex due to an increase in both the p*K*_a_ of lumenal residues and in the dipole moment of LHCII, which allows for further conformational change and clustering in the membrane. Our results suggest a mechanistic role for PsbS as a facilitator of a hydrophobic mismatch-mediated phase transition between LHCII-PsbS and its environment. This could act as the driving force to sort LHCII into photoprotective nanodomains in the thylakoid membrane. This work shows an example of the key role of the hydrophobic mismatch process in regulating membrane protein function in plants.

## Introduction

The thylakoid membrane is a complex superstructure of stacked grana membranes and interconnecting stromal lamellae and is the site of the light-dependent phase of photosynthesis. The plant thylakoid is predominantly composed of nonphosphorus glycolipids with a small population of phospholipids.^1^ Understanding the specific functions of these lipids and the dynamics of the thylakoid membrane has become an urgent matter to unravel the intricate mechanisms of photosynthetic regulation. One important regulatory process involves the fine-tuning of photosynthetic light harvesting. To attenuate the flux of excitation energy that reaches the delicate water-splitting machinery at the heart of PSII, plants have evolved a negative feedback mechanism, known as nonphotochemical quenching (NPQ), in which excess absorbed light energy can be harmlessly dissipated as heat.^2^ The major photoprotective NPQ component, known as qE, is located within the membrane-intrinsic major PSII light-harvesting complex (LHCII) and is triggered by the transthylakoid ΔpH gradient that forms as a result of photosynthetic electron transport.^3^ The allosteric modulator protein PsbS and xanthophyll pigment zeaxanthin have been shown to modulate the kinetics of the qE response.^2,4^ Reversible LHCII oligomerization and clustering in the membrane have been shown to occur in the qE state, promoted by both zeaxanthin and PsbS.^5,6^

Nevertheless, questions about how the dynamics of the thylakoid membrane, the lipids therein, and the PsbS protein itself control light harvesting and qE in LHCII remain pressing. Alongside reversible clustering of LHCII in the qE state, light-induced thylakoid membrane thinning has been observed not to correlate with ΔpH as previously proposed, but with qE itself.^7−9^ We had hypothesized that membrane thinning and lipid rearrangements could provide the driving force required to induce and reverse qE-associated LHCII clustering though a hydrophobic mismatch between the proteinaceous and lipid phases of the thylakoid membrane.^2,10^ Hydrophobic mismatch is a ubiquitous regulatory mechanism in biological membranes.^11^ Indeed, in other systems, membrane bilayer thickness has been shown to be a key modulator of membrane protein function and organization.^12^ Hydrophobic mismatch between the hydrophobic lengths of lipid and proteins within a membrane has been shown to modulate protein conformation, sorting, and aggregation in the Golgi apparatus^13^ and to direct vesicle trafficking along cell membranes.^14^ In the plant thylakoid, we here propose that to minimize the energetic unrest caused by observed changes in the hydrophobic height of the membrane, unquenched LHCII will be forced to undergo a conformational change into a more flattened, quenched state^15−17^ and will undergo self-assembly into clustered photoprotective nanodomains.^6^ In this work, we further suggest that the LHCII-PsbS complex is a key seeding point for this driving force and the observed protein rearrangements in the thylakoid that regulate photosynthesis.

## Results

**Figure 1:**
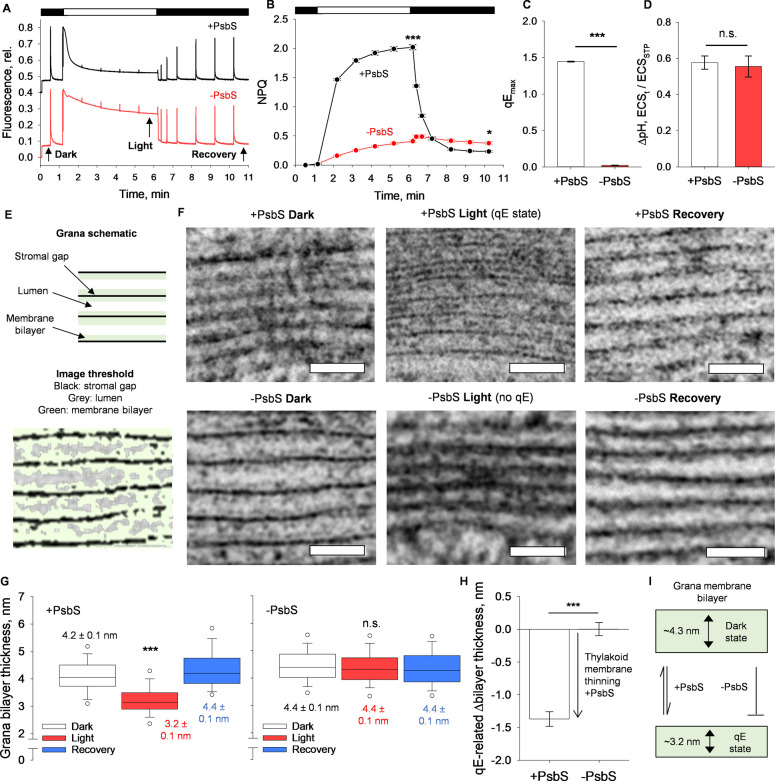
PsbS-mediated membrane thinning provides the driving force for qE *in vivo.* (A) Representative pulse-amplitude-modulation (PAM) fluorescence traces of +PsbS (black) and −PsbS (red) leaves illuminated for 5 min followed by 5 min darkness, as indicated by black and white bars above the figure. Arrows and labels indicate points of dark, light, and recovery, as referred to in other figures. (B) NPQ levels as measured through PAM fluorescence as in (A). NPQ measured as , where Fm is the maximum fluorescence after dark adaptation and Fm′ is the maximum fluorescence in the light-adapted states, each quantified with a multiple-turnover saturating pulse. Error bars represent SEM (*n* = 3). ***P < 0.001; *P < 0.05 (*n* = 3; Student’s *t* test). (C) qE calculated from PAM fluorescence traces as , where Fm″ is the maximum fluorescence after recovery from the light state and Fm′ is the maximum fluorescence in the light-adapted state. ***P < 0.001 (*n* = 3; Student’s *t* test). (D) Qualitative ΔpH levels as measured through electrochromic shift measurements. See Methods Section for more details. n.s. = no significant differences (*n* = 3; Student’s *t* test). (E) Schematic representation of the grana membrane ultrastructure as it relates to the cryo-immobilized transmission electron micrographs in (B) (upper panel). Example image of −PsbS dark with contrast highlighted in ImageJ to show areas of stromal gap (black), lumen (gray), and membrane bilayer (green) (lower panel). (F) Representative cryo-immobilized transmission electron micrographs of the stacked grana sections of the thylakoid membrane in WT (+PsbS) or npq4 (−PsbS) *Arabidopsis* leaves fixed in dark, light, or recovery states. Scale bar = 50 nm. (G) Grana bilayer thickness from micrographs of +PsbS and −PsbS leaves. Data shown are taken from independent measures of grana bilayers from at least three biological replicates (*n* = 104–154). ***P < 0.001; n.s. = no significant differences (Student’s *t* test between light and recovery states). Inset values are the average value for each data set ± SEM. (H) qE-related change in bilayer thicknesses calculated from the differences in membrane thickness between light and recovery states. ***P < 0.001. (I) Schematic representation of PsbS-induced thinning of the thylakoid bilayer. In the dark, the membrane thins from approximately 4.3 to 3.2 nm in the presence of PsbS. Without PsbS, the apparent membrane thinning response is eliminated.

To test this hypothesis, we took wild-type and PsbS-knockout *Arabidopsis thaliana* (*Arabidopsis*) plants (hereafter, +PsbS and −PsbS, respectively) and measured the dynamic changes in thylakoid membrane bilayer thickness (Figure 1F,G). In each mutant, despite an equivalent ΔpH gradient between lines, as measured through the light-to-dark transition electrochromic shift signal, the large qE response in +PsbS plants is diminished in the −PsbS condition (Figure 1A–D). Using high-pressure freezing (HPF), coupled with freeze substitution of leaf sections, we obtained a cross section of leaves to view the thylakoid ultrastructure via transmission electron microscopy (TEM). We utilized this as the primary fixation technique, instead of traditional chemical fixation, for two main reasons. Namely, the ability through this technique to better resolve the actual dimensions of the thylakoid bilayer and to reduce any artifacts that may be imposed through long chemical fixation periods.^18,19^ Through HPF coupled with external illumination, leaf sections were fixed in three states: a dark-adapted state (dark), a light-adapted qE state (light), and a postillumination dark recovery state (recovery) (Figure 1A,F; Supplementary Figure 1). TEM micrographs of the thylakoid grana, the PSII locus, show a repeating stacked structure (Figure 1E & F). The dark widths of the thylakoid membrane are 4.2 ± 0.1 nm and 4.4 ± 0.1 nm, for the + PsbS and – PsbS conditions, respectively. In the + PsbS light condition, the grana thylakoid membrane flattens to 3.2 ± 0.1 nm, as in previously published microscopy studies.^18^ This process reverses in the + PsbS recovery condition, where the thylakoid membrane has a width of 4.4 ± 0.1 nm, similar to the dark dimensions. Notably, in the absence of PsbS, the apparent membrane thinning does not occur in the thylakoid grana in the light (Figure 1F – H). Furthermore, we also chemically fixed leaf sections for TEM analysis in the light state in both conditions, although this technique has drawbacks, as stated previously,^18^ we saw similar changes in grana membrane thicknesses in that the -PsbS thylakoid membrane did not experience as much thinning in the light relative to the + PsbS condition (Supplementary Figure 1B). Thus, the light-induced thinning of the thylakoid membrane correlates well with the extent of qE and the presence of the PsbS protein itself.

Recent simulations of lipid-only thylakoid membranes have shown an approximate bilayer thickness of ∼3.0 nm.^20,21^ However, microscopy analyses of dark-adapted plant thylakoids have estimated the thylakoid grana bilayer thickness at ∼4–4.5 nm,^18,22,23^ in agreement with this study (Figure 1F–I). Therefore, it is likely that the observed *in vivo* membrane thickness depends not on the lipid phase but the proteinaceous phase of the thylakoid. Furthermore, as the thylakoid grana is densely packed with intrinsic proteins, predominantly LHCII,^6,24−26^ the membrane thinning induced by PsbS is presumably due to the qE-associated conformational change in LHCII rather than an initial change in the thickness of the lipid phase itself. The thickness of other eukaryotic cell membranes has been shown to be predominantly dependent on their protein content.^27^ This is further corroborated here by the membrane thinning response being eliminated in the PsbS-knockout mutant (Figure 1F). Indeed, loss of lutein (Lut) CD signals, FTIR measurements on LHCII trimers, and cryo-EM studies have indicated a flattening of LHCII in the qE state.^15−17^ Incorporation of LHCII into artificial proteoliposomes with acyl chain lengths shorter than that of the native thylakoid (i.e., an artificially thinner membrane) has been shown to cause LHCII to adopt a quenched conformation that promotes its clustering.^28^ Recent cryo-EM structures of LHCII in nanodisks have further shown that this flattening correlates well with a decrease in the overall fluorescence lifetime of the trimers, decreasing the distance between the terminal emitter Chlorophyll (Chl) 612 and the carotenoid Lut1 quencher acting as a quantum switch to dissipate excess energy.^17^ Thus, localized binding of PsbS to LHCII^29,30^ may act as a seed to induce this conformational change and clustering in neighboring proteins through hydrophobic mismatch, causing a phase separation in the membrane, which would act as the driving force to sort LHCII into clusters to stabilize the qE state.

However, this still raises many questions as to the molecular action of PsbS itself to induce and drive qE in LHCII. Recent course-grained molecular dynamics (MD) simulations demonstrated the involvement of specific membrane lipids in the qE mechanism.^31^ Two crucial glycolipids, the nonbilayer-forming monogalactosyldiacylglycerol (MGDG) and the bilayer-forming DGDG, constitute around 70–80% of the total lipid mass of the thylakoid.^32^ The MD simulations showed that when LHCII was exposed to a low pH environment, DGDG accumulated at the lumenal leaflet of the thylakoid around LHCII, inhibiting its transition to its quenched state. However, when LHCII formed a complex with PsbS, DGDG was blocked and thus LHCII was allowed to enter its quenched conformation and preferentially clustered in the simulated membrane.^31^ While MGDG knockdown mutants appear to have a reduced qE response,^33^ a purely genetic approach may not be straightforward due to pleiotropic effects associated with the perturbed thylakoid structure and reduced electron transport. Moreover, the biochemical validation of this hypothesis has so far remained problematic. The study of membrane proteins has often relied upon the use of membrane-disrupting nonionic detergents, such as α- or β-*n*-dodecyl-D-maltoside.^34^ These detergents replace the lipids solvating the protein, making the study of lipid–protein and weaker protein–protein interactions an issue in such cases. To address this, detergent-free isolation methodologies have been recently developed.^35^ Use of the styrene-maleic acid copolymer (SMA) allows the extraction of membrane proteins in water-soluble form while maintaining the surrounding lipid environment.^35−37^ Recent high-throughput evaluation of SMA polymers has identified approaches that allow efficient solubilization of the thylakoid.^38,39^

**Figure 2:**
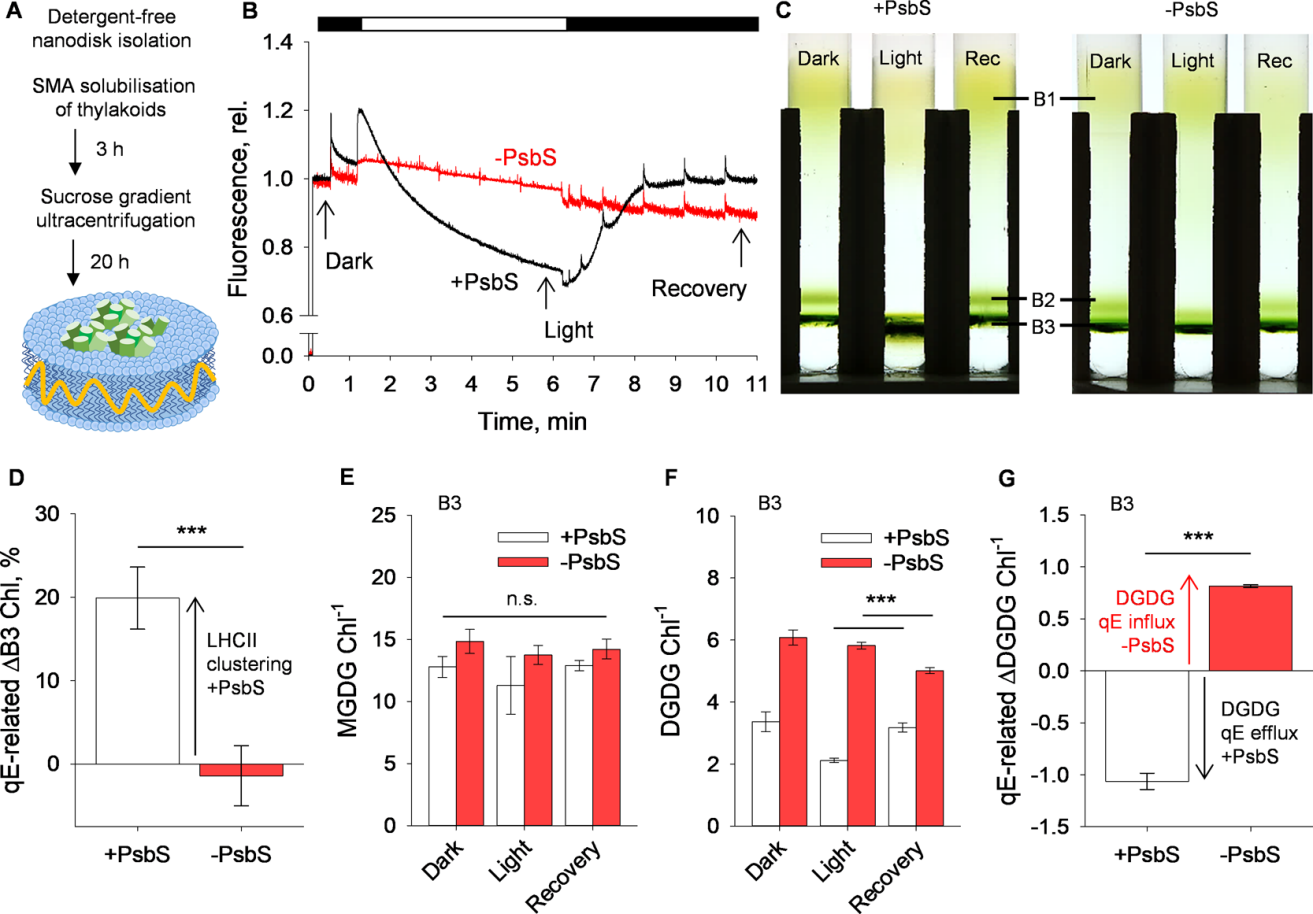
Dynamic lipid rearrangements mediated by PsbS control qE in a minimal membrane. (A) Schematic representation of nanodisk isolation from thylakoid membranes using the styrene-maleic acid (SMA) copolymer. (B) Representative PAM fluorescence traces of intact chloroplasts from lincomycin-treated minor antenna-knockout *Arabidopsis* mutant (+PsbS) and its corresponding PsbS-knockout line (−PsbS). The black bar above trace indicates periods of darkness, while the white bar indicates the illumination period at 666 μmol photons m^–2^ s^–1^. Arrows indicate dark, light, and recovery states relative to the illumination periods. (C) Sucrose density gradients of nanodisks obtained postsolubilization in dark, light, and recovery states for the + PsbS and – PsbS conditions. Three obtained bands can be seen and are highlighted here: band 1 (B1), band 2 (B2), and band 3 (B3). (D) qE-related ΔB3 chlorophyll (Chl) content quantified from absorption spectra of each band. Calculated from the solubilized Chl yields from light and recovery conditions. ***P < 0.001 (*n* = 3–4; Student’s *t* test). (E) MGDG per Chl in the isolated B3 fraction for dark, light, and recovery states for both +PsbS and −PsbS conditions. n.s. = no significant differences (*n* = 3; ANOVA). (F) DGDG per Chl in the isolated B3 fraction for dark, light, and recovery states for both +PsbS and −PsbS conditions.***P < 0.001 (*n* = 3; Student’s *t* test between light and recovery states). (G) qE-related ΔDGDG per Chl in the isolated B3 bands. Calculated from the difference between in light and recovery states for both +PsbS and −PsbS conditions. ***P < 0.001 (*n* = 3, Student’s *t* test).

To attempt to isolate LHCII clusters with SMA (Figure 2A), we utilized lincomycin-treated *Arabidopsis* minor antenna-knockout mutants (*NoM*) that constitutively overexpress the qE locus and downregulate PSII and photosystem I (PSI) reaction centers while remaining green.^3^ This system has been well characterized in *Arabidopsis*,^3,40,41^ with similar methods also employed in the green alga Chlamydomonas.^42−44^ Using this background, we further utilized a line that expresses PsbS and a PsbS-knockout mutant (hereafter, +PsbS and −PsbS, respectively). Upon illumination, +PsbS chloroplasts still showed a reversible qE response, while in the −PsbS chloroplasts, the qE response was completely absent (Figure 2B). Each line was again fixed in three qE-relevant states: “dark”, “light”, and “recovery”. Samples were then solubilized with SMA and run on sucrose gradients (Figure 2A,C), which yielded a distinct profile. Here, three well-defined bands can be seen, hereafter band 1 (B1), band 2 (B2), and band 3 (B3). Interestingly, the yield of light-B3 appeared to visibly and reversibly increase in the +PsbS condition in the light, while the yield of each band in the −PsbS condition appeared to remain static, as shown through the Chl content of each band in each condition (Figure 2C,D; Supplementary Figure 2).

Spectroscopic and biochemical analysis showed B1 likely to be free pigment, B2 likely to be a mixture of PSI and PSII core complexes, and B3 to be predominantly LHCII clusters in each light condition (Supplementary Figures 3 and 4). B3 in all conditions displays prominent 77K fluorescence emission peaks at 680 nm (F680) and 696 nm (F700), which are well-known fingerprints of LHCII, with the F700 that prominently appears under conditions where LHCII is clustered and quenched.^45−47^ However, in each B3 band, there were some far-red emission peaks at ∼715–722 nm. While such emissive states may possibly be present in pure LHCII,^45,48−50^ they may be indicative of contamination of B3 by free LHCI, which emits at this position. However, free LHCI is highly fluorescent at room temperature,^51,52^ but no LHCI-related fluorescence peak can be seen in the room-temperature spectra of B3 nor can LHCI be clearly seen in the SDS-PAGE of the sample. Yet, the Chl *a*:*b* ratio was higher than that expected for pure LHCII (Supplementary Figure 4). Thus, we cannot conclusively rule out some minor contamination of this band. However, the spectral signatures of B3 lead us to reasonably assume that the band is predominantly trimeric LHCII. This is further compounded through the lack of minor antenna in the *NoM* mutant and a large reduction in PSII and PSI core complexes in lincomycin-treated plants. SMA solubilization of the thylakoid membrane remains an exciting avenue to be explored and characterized further in future studies.^39^

As the F700 band is smaller in the −PsbS B3, it may be that the extent of antenna clustering is less than that of the +PsbS B3 (Supplementary Figure 4). Interestingly, fluorescence lifetime analysis further revealed each B3 band to be equally quenched, regardless of the condition (Supplementary Figure 4). Here, it seems that the total yield of antenna clusters is being modulated by PsbS, rather than the individual lifetime itself, in agreement with past fluorescence lifetime snapshot measurements.^53^ Both lincomycin-treated plants and *NoM* plants contain large fractions of LHCII that are both spatially and energetically uncoupled from the PSII core, relative to wild-type plants.^40,54,55^ Lincomycin treatment of *NoM* plants will undoubtedly enhance this phenotype. This is shown here, as in the dark, there is still a fraction of antenna clusters under all conditions (Figure 2C). It is likely that in the wild-type membrane, these clusters are broken up by PSII core complexes. Moreover, the presence of minor antenna complexes in wild-type membranes may act to ensure not only efficient energetic attachment to the PSII core complex but also efficient physical attachment,^55,56^ preventing LHCII clustering and aggregation under conditions where a strongly quenched qE environment may be undesirable in the plant. However, here, it is evidently shown that PsbS significantly and reversibly increases the total yield of antenna clusters in light, in agreement with electron microscopy studies on wild-type membranes. Through measurement of the Chl of the solubilizable yield of each band, here B3 reversibly increases in total yield by ∼20% in the light (Figure 2D); this increase is eliminated in the −PsbS condition. This likely reflects the increased clustering of antenna complexes in the thylakoid membrane due to the qE-related reorganization of PSII mediated by PsbS.^5,6^

To examine the lipid phase of the isolated B3 bands, lipidomic analysis was carried out on the B3 bands from both the +PsbS and −PsbS conditions. Per Chl, there were no significant differences in the MGDG content resulting from either the presence of PsbS or light treatment (Figure 2E). However, there were large differences in the DGDG content of the B3 band (Figure 2F). Furthermore, the difference between light and recovery samples shows lipid fluxes associated with the qE in the antenna clusters. While the differences in MGDG here could not be resolved (Figure 2E), the fluxes of DGDG show a change that correlates with the qE response. Here, in the +PsbS condition, there is an efflux of DGDG from the B3 band in the qE state, while in the −PsbS condition, there is an influx of DGDG (Figure 2G). This agrees with recent course-grained MD simulations where DGDG was shown to inhibit the full extent of the qE-related conformational changes in LHCII, i.e., the change from a light-harvesting to photoprotective state.^31^ The removal of DGDG from LHCII-PsbS has been suggested to increase the mobility of such complexes in the membrane, as seen in fluorescence recovery measurements of chloroplasts.^5^ DGDG further appears to be a vital boundary lipid associated with LHCII, facilitating its 2D crystallization^57^ and conferring higher thermal stability to the complex.^58^ Conversely, the decreased DGDG content around LHCII, often via increased MGDG concentrations, has been proposed to alter the mechanical stability of LHCII^59^ and to modulate the lateral hydrostatic membrane pressure about LHCII.^59,60^ Indeed, increasing MGDG concentrations and thus decreasing DGDG concentrations in LHCII proteoliposomes have shown shorter fluorescence lifetimes at neutral pH,^60^ indicating the role of these lipid fluxes in directing LHCII from a light-harvesting to a photoprotective, quenched state.

**Figure 3:**
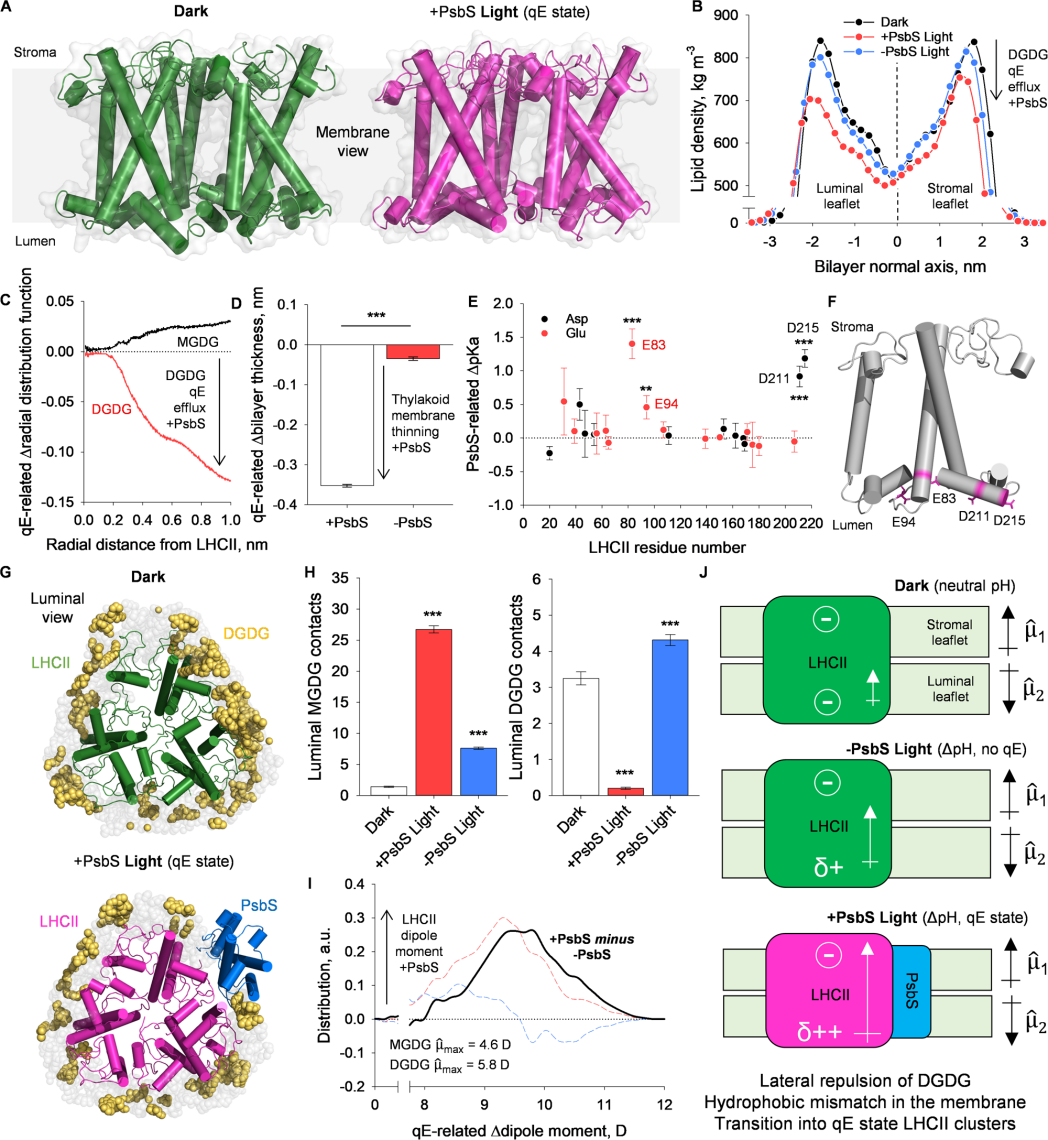
Molecular role of PsbS, lipid rearrangements, and thylakoid membrane thinning shown through all-atom MD simulations and Markov state modeling. (A) Structures of an LHCII trimer in a dark and +PsbS light state calculated from Markov state model projections of all-atom models. Structures were visualized in PyMOL. (B) Lipid density around the LHCII trimer in the dark, +PsbS light, and −PsbS light states along the axis of the membrane normal. (C) qE-related Δradial distribution function for the MGDG and DGDG lipids in the presence of PsbS up to 1 nm away from the center of the LHCII trimer. Calculated from the difference between dark models and +PsbS light models. (D) Average qE-related Δbilayer thickness for both +PsbS and −PsbS conditions. Calculated from the difference between dark and light states for both conditions. ***P < 0.001 (*n* = 3; Student’s *t* test). (E) PsbS-related Δp*K*_a_ for the aspartate (Asp; D) and glutamate (Glu; E) residues of LHCII. Calculated from differences between dark and +PsbS light models. ***P < 0.001; **P < 0.01 (*n* = 9; Student’s *t* test between dark and +PsbS light conditions). (F) Schematic model of the LHCII monomer with the residues that experience a significant shift in p*K*_a_ with PsbS highlighted. Structure visualized in PyMOL. (G) Luminal view of an all-atom model of the LHCII trimer in a membrane environment in a dark state and a +PsbS light state. DGDG lipids are shown in gold. Structures were visualized in PyMOL. (H) Luminal MGDG and DGDG contacts between the lipids and the LHCII trimer. Contacts measured as an atomic distance <0.6 nm between any part of the lipid molecule and an Asp or Glu residue of LHCII. (*n* = 3, Student’s *t* test between the dark state and respective light states). (I) qE-related Δdipole moment of the LHCII trimer in both +PsbS and −PsbS conditions. Difference taken between dark and +PsbS light or −PsbS light conditions. Inset values are maximum calculated dipole moments for bulk MGDG and DGDG lipids. (J) Schematic representation of dipole moments of LHCII relative to the thylakoid membrane. LHCII protonation with bound PsbS increases the dipole moment of the LHCII trimers that preferentially expels the DGDG lipid phase.

To gain an atomistic view of the molecular action of PsbS in the induction of membrane thinning and lipid rearrangements in qE, we performed all-atom MD simulations on LHCII and PsbS in different states. Here, we intended to clarify how and why LHCII-PsbS expels surrounding lipids and how it transitions into its quenched and flattened conformation. We further aimed to gain insights into the causality and sequence of events that were seen in the prior experimental microscopy and biochemical results. To do this, LHCII was placed in a simulated membrane with a lipid composition representative of the *in vivo* thylakoid membrane.^1^ Three states were modeled: a “dark” state; a “+PsbS light” state; and a “–PsbS light” state. The dark state represents LHCII in the membrane at neutral pH, while the light states simulate the effects of the ΔpH gradient with and without PsbS (Figure 3A). All-atom and Markov state model structures here show high similarity to the recently solved cryo-EM structures of dark and light states of LHCII in nanodisks (Figure 3A).^17^ The conformational change from the light-harvesting to the energy-dissipative state involves the retraction of lumenal helices D and E into the hydrophobic core of LHCII and the decrease in the crossing angle between the two transmembrane helices A and B.^17,61^ In our all-atom model, this structural transition is coupled with an alteration in the lipid environment about LHCII. Under the +PsbS light condition, there is an overall decrease in the lipid density through the bilayer normal around LHCII, with a greater decrease in density in the lumenal thylakoid leaflet (Figure 3B). This reflects a DGDG efflux in the qE condition, as shown in the radial distribution of lipids around LHCII (Figure 3C,G). This DGDG efflux during qE appears to be coupled to a smaller influx of MGDG (Figure 3C). These lipid rearrangements and conformational changes of LHCII are further coupled to a PsbS-dependent vertical thinning of the bilayer (Figure 3D), as in the microscopy results in Figure 1. It has been previously proposed that the action of PsbS during qE allows LHCII to become more sensitive to the ΔpH gradient.^62^ Here, quantification of the p*K*_a_ values of LHCII aspartate (Asp; D) and glutamate (Glu; E) residues show that PsbS binding shifts the p*K*_a_ of E83, E94, D211, and D215 toward more neutral values (Figure 3E,F). Indeed, these changes are concomitant with an alteration in the interpigment distances in the terminal emitter region of LHCII in the presence of PsbS (Supplementary Figure 5E). In our simulations, we record a decrease in the mean distance between Chla 612 and the Lutein-1 (Lut1) at the low pH +PsbS condition, relative to both the low pH −PsbS and neutral pH conditions. Thus, in the presence of PsbS, this change in distance should facilitate a faster exciton energy transfer from the Chl-a Qy to the short-lived S1 or S* state of Lut-1, promoting quenching. This highlights the role of PsbS as an allosteric modulator that allows LHCII to transition into its quenched state under lower ΔpH conditions.

However, while the PsbS-dependent photoprotective switch in LHCII appears to involve a thinning of the complex and rearrangements of surrounding lipids, questions remain on how this process occurs on a molecular level. To measure the impact of the apparent DGDG stripping on LHCII, we further quantified the change in contacts between DGDG and MGDG and LHCII in the lumenal leaflet, where larger changes occur (Figure 3G,H). This is a measure of the amount of contact points between a lipid molecule and an LHCII Asp or Glu residue (<0.6 nm), rather than the number of lipids within a particular unit distance of LHCII (Figure 3B,C). In each light condition, there is a large increase in MGDG contacts relative to the dark condition, with the largest increase seen in the +PsbS condition. Given that the actual amount of MGDG lipids does not greatly increase in the light states (Figure 3C), these data imply that MGDG lipids associate more strongly with LHCII under low pH conditions. While this could be partially due to the DGDG stripping in the +PsbS condition, without PsbS, there is a simultaneous increase in both lumenal MGDG and DGDG contacts. However, this still raises questions as to why DGDG is stripped in the +PsbS light condition. To answer this, we further quantified the changes in the LHCII dipole moment in each condition (Figure 3I). Through this, we found a pronounced ΔpH-related and PsbS-dependent dipole moment increase at ∼10 D. Given that the bulk DGDG content exerts a higher dipole moment compared to MGDG, there will likely be a larger LHCII-DGDG repulsion. The dipole moment of LHCII is further increased by its protonation, which is largely dependent on PsbS (Figure 3I,J). Thus, it is likely that the fact that protonated LHCII-PsbS will strongly repel DGDG largely dictates the lipid rearrangements associated with qE. This, coupled with the LHCII flattening associated with its conformational change into its photoprotective state, is a strong candidate as the initial point of hydrophobic mismatch in the thylakoid membrane and likely acts as the initial photoprotective seed to induce further quenching in surrounding LHCII trimers that do not have PsbS bound.

## Discussion

**Figure 4:**
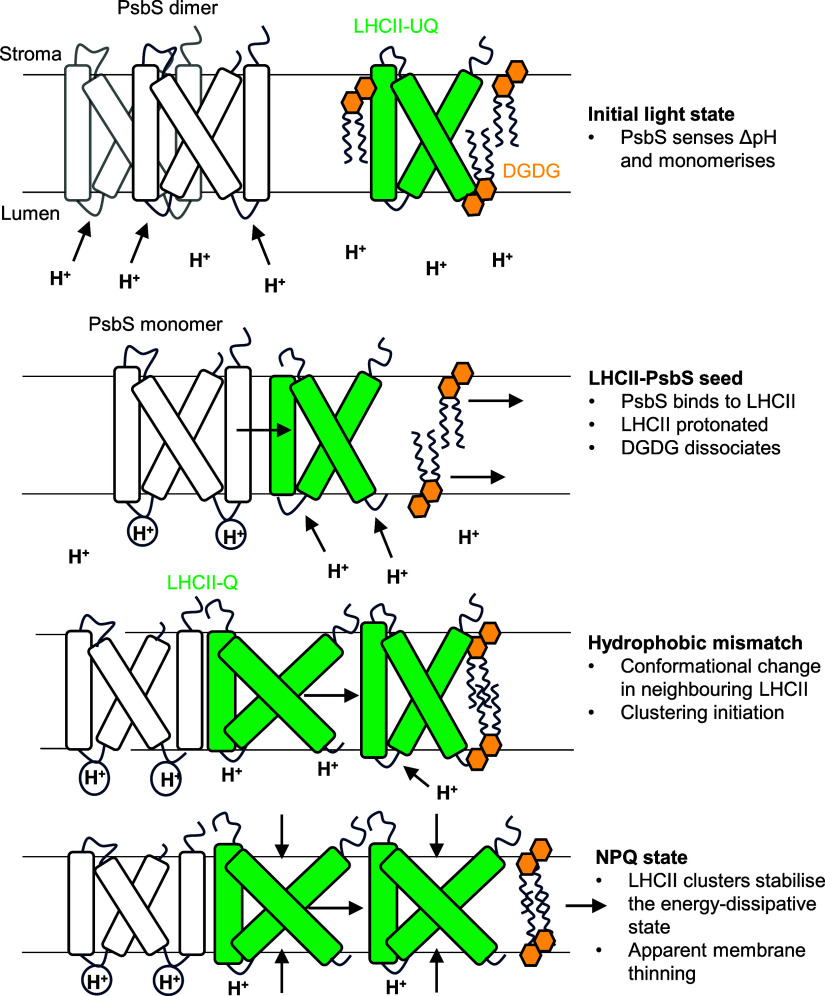
Schematic model of the hydrophobic mismatch hypothesis as it relates to NPQ in the thylakoid membrane. In the initial light state, ΔpH forms across the thylakoid due to photosynthetic electron transport and is sensed initially by the PsbS dimer. PsbS monomerises and binds to the unquenched LHCII antenna (LHCII-UQ), and DGDG is preferentially expelled from this initial seed complex. Concomitantly, LHCII is protonated and undergoes a conformational change to its quenched state (LHCII-Q). This causes a hydrophobic mismatch in the thylakoid membrane as the PsbS-LHCII complex is flatter than its unquenched form. This exerts a pressure on surrounding LHCII-UQ allowing it to become protonated and enter its quenched conformation. As a result, the thylakoid membrane rearranges and LHCII clusters stabilize the energy-dissipative state. More details and references can be found in the discussion section of the main text.

The data in this study provide a mechanistic hypothesis on the molecular role of PsbS in qE (Figure 4). Upon the formation of the transthylakoid ΔpH, PsbS is more readily protonated than LHCII.^62,63^ PsbS then has been shown to monomerise^64^ and bind to LHCII,^29,30^ causing LHCII to become more sensitive to the ΔpH gradient (Figure 3E).^62^ This would cause further conformational change in LHCII, which has been shown to be associated with flattening of the LHCII complex in the membrane.^15−17^ It is worth noting that the low pH-induced conformational change in PsbS also involves a flattening in the plane of the membrane.^63^ This change in both LHCII and PsbS can be indirectly observed here though PsbS-induced thinning of the thylakoid grana bilayer in light (Figure 1). PsbS’ binding to LHCII, LHCII’s sensitivity to the ΔpH gradient, and the apparent membrane thinning have all been independently shown to be further enhanced by the presence of zeaxanthin.^9,29,30,62^ The flattening of LHCII is expected to cause the structural perturbation of Chl 612 and Lut1 that opens the energy-dissipative channel.^17,65^ Without PsbS, the effects of ΔpH on LHCII has been shown to be counteracted by DGDG accumulating around LHCII, partially inhibiting the flattening of the complex.^31^ This is supported by data in this study showing a lack of ΔpH-induced LHCII clustering (Figure 2C) and a DGDG influx in the absence of PsbS and qE (Figures 2G and 3H). In the presence of PsbS, LHCII clustering increases in the light as these fractions are further stripped of DGDG. It is likely that PsbS is playing a role in this stripping process as suggested here and previously.^31^ It appears that the quenched conformation of the LHCII-PsbS complex electrostatically repels the DGDG lipid due to changes in the dipole moment of LHCII-PsbS at low pH (Figure 3I). Indeed, work in proteoliposomes has shown that increased concentrations of MGDG around LHCII favors energy-dissipative states due to the ability of MGDG to modulate hydrostatic membrane pressure of the LHCII complex.^60^ While the lipidomics could not resolve any changes in MGDG around LHCII, the MD simulations implied a small influx of MGDG in the qE state, coupled with the much larger DGDG stripping (Figure 3C). Due to the substoichiometric presence of PsbS in the membrane,^66^ the initial LHCII-PsbS interaction may act as a seeding complex for further LHCII clustering by hydrophobic mismatch, in that the flattening of LHCII will cause thermodynamic unrest in neighboring proteins causing further conformational change, LHCII clustering, and, ultimately, further stabilization of the qE state (Figure 4). Upon cessation of illumination and relaxation of the ΔpH gradient, this process appears to be fully reversible, a hallmark of qE.

We suggest a mechanism of the photosynthetic regulator PsbS as a mediator of dynamic lipid rearrangements that allows a conformational change in LHCII that involves flattening of the complex. This causes a hydrophobic mismatch in the thylakoid membrane between the quenched conformation of LHCII and its environment, which in turn induces a phase transition in the thylakoid membrane that sorts the quenched antenna into photoprotective nanodomains. The identification of the mechanism of PsbS will allow further manipulation of plants ultimately to improve crop yields in highly variable light environments.^67,68^

## Supporting Information Available

Description of all materials and methods, supplementary micrographs and quantification, chlorophyll distributions, absorption and fluorescence spectra, SDS-PAGE, expanded computational analyses (PDF)
