# Supplementary material for: Hydrophobic Mismatch in the Thylakoid Membrane Regulates Photosynthetic Light Harvesting
Source: J Am Chem Soc. 2024 May 17;146(21):14905–14. doi: 10.1021/jacs.4c05220 (PMC11140739; doi:10.1021/jacs.4c05220)
Supplement: Supplementary file 1 — ja4c05220_si_001.pdf [file ja4c05220_si_001.pdf]

Supporting Information

Hydrophobic mismatch in the thylakoid membrane regulates photosynthetic light harvesting

Sam Wilson[1#], Charlea D. Clarke[1], M. Alejandra Carbajal[2], Roberto Buccafusca[3], Roland A. Fleck[2], Vangelis Daskalakis[4] and Alexander V. Ruban[1**]

[1]Department of Biochemistry, School of Biological and Behavioural Sciences, Queen Mary University of London, London, E1 4NS, United Kingdom

[2]Centre for Ultrastructural Imaging, King's College London, London, SE1 1UL, United Kingdom

[3]Department of Chemistry, School of Physical and Chemical Sciences, Queen Mary University of London, London, E1 4NS, United Kingdom

[4]Department of Chemical Engineering, School of Engineering, University of Patras, Patras, 26504, Greece

[#]current address: Division of Environmental Photobiology, National Institute for Basic Biology, Okazaki, 444-0867, Japan

*corresponding author: a.ruban@qmul.ac.uk (A.V.R.)

Methods

Plant material and growth conditions

Arabidopsis seeds were sterilised in a medium containing 50% (v/v) ethanol and 0.1% (v/v) Triton X-100 and stored for 48 h at 4 °C before washing and storage at 4°C for 48 h prior to sowing on a 6:6:1 Levington M3 compost / John Innes No. 3 soil / Perlite mixture (Scotts, UK).

Wild type Arabidopsis (Col-0) and the PsbS-knockout mutant (*npq4*)[1] were used for the microscopy studies. Whilst Arabidopsis mutants lacking minor antenna (*NoM*)[2] and lacking both minor antenna and PsbS (*NoM npq4*)[3] were used for lincomycin treatment, biochemical, and lipidomic analysis. These were treated with lincomycin as previously described[4–6] at 6 weeks old for 2 weeks until Fv/Fm was approximately 0.2 – 0.3. Plants were not etiolated and remained green during treatment, despite a diminished growth rate. All plants were grown at 150 µmol photons m^{-2} s^{-1} with a 10 h photoperiod at 22°C. Plants were grown in a Percival AR-75L3 plant growth cabinet (Percival Scientific Inc., USA), equipped with Phillips MASTER TL-D Super 80 36 W/840 bulbs, which emit a cool white light (Koninklijke Philips N.V., Netherlands). Plants were dark-adapted overnight prior to further experiments. Chl was quantified according to previously published protocols[7].

Isolation of protoplasts and chloroplasts

Protoplasts were isolated as previously described[4,8]. Leaves were detached and the abaxial epidermal layer was gently removed with adhesive tape. Stripped leaves were then floated on a solution containing 0.4 M D-mannitol, 20 mM KCl, 10 mM $CaCl_2$, 20 mM MES (pH 5.6), and 0.1% BSA (protoplast buffer), with 1.5% cellulose Onuzuka R-10 and 0.4% maceroenzyme R-10 (Serva, Germany), for 1 h. The solution was centrifuged at 100 x *g* for 3 min at 4°C, washed with the protoplast buffer, and centrifuged again at 100 x *g* for 3 min at 4°C. The obtained protoplasts were suspended in a resuspension medium containing 0.33 M sorbitol, 5 mM $MgCl_2$, 10 mM KCl, 2.5 mM EDTA, and 50 mM HEPES (pH 7.6). Protoplasts were stored in the dark on ice prior to further experiments. For intact chloroplasts, protoplasts were osmotically broken *in situ* immediately prior to experiments, as previously described [4].

PAM fluorescence methods

For measurement of NPQ induction on leaves, measurements were undertaken on a DUAL-PAM-100 fluorescence measuring system (Walz, Germany). Measuring light

intensity was < 10 µmol photons m^{-2} s^{-1}, saturating pulses were 4000 µmol photons m^{-2} s^{-1}, and the actinic light intensity was 812 µmol photons m^{-2} s^{-1}. Leaves were illuminated for 5 min, followed by 5 min darkness.

Prior to each measurement on isolated organelles, protoplasts were osmotically broken *in situ* using a break medium containing 5 mM $MgCl_2$, 10 mM KCl, 2.5 mM EDTA, 50 mM HEPES (pH 7.6) in a cuvette for 30 s whilst stirring, before osmotic balance was returned using a reaction buffer containing 0.66 M sorbitol, 5 mM KCl, 20 mM sodium citrate, 10 mM EDTA, 10 mM $NaHCO_3$, 20 mM HEPES (pH 8.0), and 0.1% BSA. At 22°C, the mixture of these two buffers gave an initial reaction pH of 7.8. Chl fluorescence induction was undertaken on a DUAL-PAM-100 fluorescence measuring system (Walz, Germany). Here, 5 min of illumination at 666 µmol photons m^{-2} s^{-1} were applied to the sample followed by 5 min darkness. The measuring light intensity was < 10 µmol photons m^{-2} s^{-1}, and the saturating pulse intensity was 4000 µmol photons m^{-2} s^{-1}. Samples were fixed in the light through acidification of the bulk buffer to pH 5.5, after which no recovery of Fm was observed. After light treatment, samples were stored on ice in the dark prior to further solubilisation.

SMA copolymer-mediated solubilisation of thylakoid membranes

SMA copolymer-mediated solubilisation of thylakoid membranes was adapted from previously published protocols[9–12]. SMALP 502-E (Orbiscope, Netherlands; also published as SMA 1440) was utilised in this study as its butoxyethanol functional group was shown to be key in ensuring a high solubilisation yield when used with plant thylakoid membranes[11]. Plant membranes fixed in dark and recovery states were solubilised in a 20 mM HEPES (pH 7.8) buffer, whilst light states were solubilised in a 20 mM MES (pH 5.5) buffer, each containing 2% SMA (v/v). SMALP 502-E has a styrene-to-maleic acid ratio of 1.7, which has been previously shown to be stable from pH 4.0 to 9.0, with no alteration in basal solubilisation efficacy or polymer stability[13]. Material corresponding to 500 µg total Chl was solubilised in 1 mL buffer for 3 h in a 25°C water bath. To remove unsolubilised material, membranes were centrifuged for 5 min at 14,000 RPM.

Sucrose gradient ultracentrifugation

Seven-step exponential sucrose gradients were performed as previously described, with some minor modifications[14]. Solubilised protein was loaded on to a 0.15 M to 1 M sucrose gradients that had a 2 M sucrose cushion. These were then centrifuged for

18 – 20 h at 40,000 rpm at 4°C. Bands were then harvested and either flash-frozen in liquid nitrogen or stored on ice until further experiments.

SDS-PAGE

SDS−PAGE experiments were performed as previously described[15]. 1.5 µg total Chl was loaded per lane of thylakoid membrane sample, whilst 20 µL of the sucrose gradient band of interest was also loaded. These were run on 12% polyacrylamide gels.

Absorption spectroscopy

For measurements of ΔpH on leaves, electrochromic shift measurements were undertaken as previously described [16]. These were done so on the Walz DUAL-PAM-100 (Walz, Germany) and its P515/535 emitter-detector modules[17], with the measuring light set to a frequency of 1,000 Hz. To calibrate each measurement to account for varying leaf thickness and chlorophyll content, the ECS signal wavelengths were balanced using the inbuilt software and normalised to the ECS signal from a single-turnover pulse. Leaves were illuminated for 5 min at 812 µmol photons m^{-2} s^{-1}, with the normalised magnitude of the light-dark transition in the ECS signal taken to be equivalent to the steady-state ΔpH[16].

Room-temperature absorption spectra were recorded on samples at a Q_y absorption maximum of 0.5 on a modernized Aminco DW-2000 UV−vis spectrophotometer (Olis Inc., USA), with an x-axis resolution of 1 nm. The optical path length was 1 cm. Spectra were normalized at their absolute maximum in the Soret region, unless otherwise stated. Absorption spectra were used to calculate the relative distribution of Chl molecules in the bands harvested from sucrose density gradients as

$$[Chl] = \int_{600}^{750} A(\lambda) \cdot d\lambda \cdot V \cdot D$$

where, $\int_{600}^{750} \mathrm{A}(\lambda) \cdot \mathrm{d}\lambda$ is the integral of the absorption as a function of wavelength, V is the total volume of the harvested band, and D is the dilution factor applied to the sample prior to measurement of each respective spectrum[18].

Steady state fluorescence spectroscopy

Steady state fluorescence spectra were recorded for samples at a Q_y absorption maximum of 0.5 using a FluoroMax-3 spectrofluorimeter (HORIBA Jobin Yvon, France) equipped with a cryostat cooled by liquid nitrogen. For emission spectra, samples were excited at 436 nm and emission was detected between 600 and 800

nm, with the detector defined by a long-pass filter with a sharp cutoff at 650 nm. An integration time of 0.1 s was used to reduce the noise level. Five scans were captured for each spectrum and subsequently averaged. Spectral correction was applied within the FluorEssence software, according to the manufacturer's specifications (HORIBA Jobin Yvon, France).

Time-correlated single photon counting

Time-correlated single-photon counting (FluoTime 200 Fluorimeter, PicoQuant, Germany) measurements were performed as previously described[16]. Samples were excited at 468 nm using a 0.6 mW laser diode at a 20 MHz repetition rate, with fluorescence detected at 680 nm with a 2 nm slit width. The optical path length was 1 cm. All samples were measured at a Q_y absorption maximum of 0.5. This setup has been previously demonstrated to have a negligible probability of singlet−singlet annihilation artifacts[19]. These data were analysed using the FluoFit software (PicoQuant, Germany), with the χ^2 parameter and autocorrelation functions used to assess the quality of the fit. Average lifetimes were calculated as described previously[20].

Quantification of MGDG and DGDG by LC-MS

Initially, galactolipids were first extracted from the sucrose gradient fraction. For this 8 mL of ice-cold ethanol-diethyl ester was added at a ratio on 3:2 (v/v) relative to the sample and was incubated at -20°C overnight. Following a brief centrifugation, the organic phase was transferred to a fresh glass conical tube and dried under a stream of N_2. The lipid film was then dissolved in 100 mL of a 9:1 (v/v) solution of chloroform and methanol. Each sample was then further diluted five-fold in a 2:1:1 solution of isopropyl alcohol, acetonitrile, and water, and was spiked with two internal standards, 100 nM hydroxyl-MGDG and 500 nM hydroxyl-DGDG. 5 µL of each sample was analysed by LC-MS.

Extracted galactolipids were separated using a 2.1 x 50 mm UPLC BEH C18 1.7 µm column (Waters, USA) kept at 65°C on an AQUITY UPLC system (Waters, USA) coupled to a Synapt G2Si High Resolution Mass Spectrometer (Waters, USA). The mobile phases consisted of (A) 5% (v/v) isopropyl alcohol in water containing 0.1% (v/v) formic acid as a modifier and (B) 5% (v/v) isopropyl alcohol in acetonitrile acidified with 0.1% (v/v) formic acid. The gradient programme followed 0 – 2 min 50% B, 2 – 12 min 50% B to 92% B, and kept for an addition 10 min at 92% B. The remainder percentages were of A.

The Synapt G2Si MS was operated in positive electrospray mode with the capillary voltage set to 3.00 kV. N_2 gas flow rates were fixed with a cone gas flow of 25 L/h and a desolvation gas flow of 900 L/h. A source temperature of 125°C and a desolvation temperature of 350°C were applied. A relative quantification approach was followed to calculate the concentration of both MGDG and DGDG, using the signals of the hydroxylated standards. UNIFI 1.9 software (Waters, USA) was used for data processing, to correlate the responses of all targeted lipids with the concentration of the two standards.

Electron microscopy analysis

Sections of wild type and *npq4* Arabidopsis leaves were removed from the plants and vacuum infiltrated with the cryo-protectant 1-hexadecene, as previously described[21,22]. Leaf material was then illuminated *in situ* with a Schott KL 2500 LED lamp defined by a long-pass filter (sharp cut-off at 640 nm). The light intensity that reached the leaf was approximately 800 µmol photons m^{-2} s^{-1}. Leaves were fixed in either a dark state (dark-adapted overnight), a light-adapted state (5 min illumination), and a recovery state (5 min illumination, followed by 5 min dark). Leaves were cut with a biopsy punch and cryo-immobilized using a high-pressure freezer, Leica EM ICE (Leica Microsystems, Austria). The light samples were frozen within 5 s after the cessation of illumination to ensure the light state was not able to relax. Samples were subsequently freeze substituted using a Leica EM AFS2 (Leica Microsystems, Austria) in dry acetone containing 0.2% (v/v) uranyl acetate, 1% (v/v) osmium tetroxide and 2% distilled water for 72 h at -90°C and warmed to 0°C over a period of 48 h. After rinsing several times with dry acetone, the samples were infiltrated and embedded in Spurr's resin (TAAB Laboratories, UK). Ultrathin sections (70 nm) were cut using an UC 7 ultramicrotome (Leica Microsystems, Austria), mounted on carbon support film copper grids (GS 2x1) and post-stained with uranyl-free stain and Reynold's lead citrate. Samples were examined using a JEOL JEM-1400Flash TEM operated at 80 kV and fitted with a 2MP JEOL Matataki camera (JEOL, Japan). Sample quality was validated according to the criteria set out by Li et al., 2020b. Images were further analysed as previously described[21,23], using the ImageJ software package[24]. Chemically fixed samples in the Supplementary Fig. 1 were prepared as previously described[25].

Molecular dynamics simulations and analysis

Model coordinates The crystal structure of the major LHCII from spinach (PDB: 1rwt; chains C, E and H)[26] was used for the initial coordinates to build the LHCII trimer model. The Amber ff14sb force field[27] was employed for the polypeptide chains. The carotenoids lutein, neoxanthin, and violaxanthin were described by Amber ad-hoc parameters developed by the group of Prof. B. Mennucci in University of Pisa and provided in ref[28]. The Chl a/b molecules were based on also previously published parameters as described in ref[29] The LHCII-PsbS complex orientation was built with only one PsbS monomer interacting with the LHCII trimer in the orientation described in refs[30,31]. Initial PsbS coordinates were taken from the PsbS crystal structure (chain A)[32]. The all-atom models, as defined previously, were embedded in a membrane patch of native thylakoid lipids described by Amber compatible force field[31,33]. The initial membrane model was constructed using Packmol (https://m3g.github.io/packmol/) and had the following composition: 512 lipids (220 MGDG, 129 DGDG, 129 SQDG, and 34 PG), 22,000 water molecules and sodium ions for neutralization. The membrane patch was energy minimized by the steepest descend algorithm (1000 steps) and it was subsequently equilibrated for 250 ns at 298K (with the same parameters and pressure-temperature couplings as for the MD production runs described below). The ad-hoc Amber 03 – compatible force field parameters for the lipids were provided by the group of Prof. D. Pantazis in Max-Planck-Gesellschaft and were parametrized as described in detail elsewhere in a combination of GLYCAM06 for the heads and Slipids for the lipid tails[34] The option -membed in Gromacs 2020.5 was used to embed the LHCII trimer within the equilibrated membrane patch. After the LHCII was integrated into the membrane patch waters were removed and the content in thylakoid lipids was adjusted to 45% mono-galactosyl-diacyl-glycerol (MGDG), 25% digalactosyl-diacyl-glycerol (DGDG), 25% sulpho-quinovosyl-diacyl-glycerol (SQDG) and 5% phosphatidyl-glycerol (PG) for consistency among all models. The thylakoid membrane was thus enriched in MGDG-DGDG lipid content at around 70% and re-hydrated by 24000 TIP3P water molecules (lipid-water ratio at ~68.6)[35]. The models contained 150 mM KCl, with a surplus of K^+ ions to neutralize both the protein charges and the negatively charged thylakoid lipids in each system. Before neutralization systems had negative charges of -144 (neutral pH), -123 (low pH –PsbS), and -109 (low pH +PsbS). The LHCII-only models contain around 137k atoms (9.8k for the protein residues, 9.6k for co-factors, 70.6k for water molecules and ions, 46.6k for lipids), the LHCII-PsbS models contain around 132k

atoms (12.3k for the protein residues, 9.6k for co-factors, 70.2k for water and ions, 40k for the lipids). The equilibrated unit cell dimensions of each model were roughly 11.6 x 11.6 x 9.6 nm^3 in dimensions.

Residue protonation states The interaction of zeaxanthin and PsbS with LHCII can alter the pKa values of lumen-exposed residues, so that LHCII can switch to the dissipating state at the physiological pH value of acidified lumen (5.5-5.8)[36]. Given a sufficiently high trans-thylakoid membrane ΔpH (i.e. lumen pH acidification to ~4.5)[37], even in the absence of PsbS or zeaxanthin[36–39], LHCII is able to transition to the energy-dissipative state. Our approach combines the experimental findings[40–42] of possible protonable residues at the lumen-exposed side of LHCII[43–45], along with the computational PROPKA method[46,47] and the effect of a proposed LHC-PsbS interaction on the residue pKa values[48]. This has led to our choice of protonating the majority of lumen exposed major LHCII residues (E83, E94, E107, and E207 and D111, D211, and D215) for the pH 5.5 models. These Glu-Asp residues are kept deprotonated for the neutral pH models. For PsbS, residues E20, E69, E173 and D21 were treated as protonated for the low pH LHCII-PsbS complex model. These latter residues of PsbS have labile protons, as suggested in a recent study[49].

The referred residues in the lumenal side of the LHCII trimer/PsbS were kept constantly protonated for the low pH models, whereas constantly deprotonated for the neutral pH models. The stroma-exposed residues were treated constantly deprotonated at all models, to simulate low transthylakoid membrane ΔpH (ΔpH ~0 light harvesting) and enhanced ΔpH (~1.5 quenched states). Thus, pH was indirectly simulated by employing constant protonation states of key residues.

Model equilibration and production runs Based on published protocols, all models were relaxed and equilibrated with gradual removal of constraints on the protein backbone-heavy atoms[31]. Briefly, the equilibration steps contained:

(1) a steepest descend energy minimization with a tolerance of 0.5 kJ/mol for 1000 steps.

(2) constant density and temperature (nVT) Brownian dynamics at 100 K for 50 ps that employ the Berendsen thermostat with a temperature coupling constant at 1.0 fs.

(3) A short constant density (nVT) run for 100 ps. The weak coupling Berendsen thermostat at 100K was employed with a coupling constant of 0.1 ps (temperature).

(4) A short constant pressure (nPT) run for 100 ps. The weak coupling Berendsen thermostat and barostat[27] at 100 K were employed with coupling constants of 0.1 ps (temperature) and semi-isotropic 50.0 ps coupling (pressure) with a compressibility of $4.6x10^{-5}$ (x-y dimension) and $4.5x10^{-5}$ (z dimension).

(5) Heating from 250 K to 298 K in a constant pressure ensemble (nPT) for 2 ns, employing the v-rescale thermostat[28] and Berendsen barostat[27], with time coupling constants of 0.1 ps for the temperature and 2.0 ps for the pressure. For this we remove also all but the Ca-atom protein position restraints.

(6) Equilibration at 298 K (0.1 ps temperature coupling constant) for 5 ns (nPT, 1 atm, 2.0 ps coupling constant for pressure).

(7) Two equilibration steps at 298 K (0.5 ps temperature coupling constant) for 5ns (nPT, 1 atm, 2.0 ps coupling constant for pressure) with different constraints on Ca-atoms.

(8) Equilibration at 298 K (0.5 ps temperature coupling constant) for 10 ns (nPT, 1 atm, 2.0 ps coupling constant for pressure)

The barostats – thermostats employed for steps 6-8 were the same as in the production trajectories that follow. Gradual relaxation of the constraints on protein heavy atoms (from 10^4 in steps-1-3 to 10^3 kJ/mol/nm^2 in step-4) and Ca atoms (from 10^3 in step-5, to 10^2 in step-6, 10 and 1 for the two runs in step-7, 0 kJ/mol/nm^2 in step-8) for around 30 ns with a time step of 1.0 fs (steps 2-4) and 2.0 fs (steps 5-8).

Classical MD simulations were run for further equilibration for 500 ns per different system-model. Newton's equations of motion were integrated with a time step of 2.0 fs. The leapfrog integrator in GROMACS 2020.5 was employed[50]. The production runs have been performed in the constant pressure nPT ensemble with semi-isotropic couplings in the x-y membrane plane and in the z-direction (compressibility at 4.5 x 10^{-5}). Moreover, the van der Waals interactions were smoothly switched to zero between 1.0 - 1.2 nm with the Verlet cut-off scheme. Short-range electrostatic

interactions were truncated at 1.2 nm and long-range contributions were computed within the Particle-Mesh-Ewald (PME) approximation[51]. All hydrogen-heavy atom bond lengths were constrained employing the LINCS algorithm[52]. The v-rescale thermostat is employed[53] (temperature coupling constant = 0.5) and the Parrinello-Rahman barostat[54] (1 atm; pressure coupling constant = 2.0).

After the extended equilibration of the models by classical MD, the replica exchange (RE) enhanced sampling method with solute tempering (REST2)[55] was used in three independent RE runs per each system (neutral pH, low pH, or low pH +PsbS). The GROMACS 2020.5 engine patched with PLUMED 2.7 was employed[56]. The LHCII trimer was considered as the solute. Ten replicas were considered for each RE run for 500 ns each at the equivalent temperatures of 298, 306, 314, 322, 330, 339, 347, 356, 366, 375 K. This totals a 45μs production simulation time (3 runs x 3 systems x 10 replicas x 0.5 μs). An exchange attempt was performed every 1000 steps (2 ps) with an acceptance rate of 9 - 15% between replicas. The first ~200 ns from each trajectory were considered as further equilibration (without exchanges) and the analysis was only performed after exchange was initiated and for the final ~300 ns of each demuxed trajectory at 298K. As three independent runs were performed per case, the actual data analysed refer to ~900ns per case (3 x ~300ns; neutral pH, low pH or low pH + PsbS). Structures were collected every 1.0 ns for all the trajectories.

References

(1) Li, X.-P.; Björkman, O.; Shih, C.; Grossman, A. R.; Rosenquist, M.; Jansson, S.; Niyogi, K. K. A Pigment-Binding Protein Essential for Regulation of Photosynthetic Light Harvesting. *Nature* **2000**, *403* (6768), 391–395. https://doi.org/10.1038/35000131.

(2) Dall'Osto, L.; Ünlü, C.; Cazzaniga, S.; van Amerongen, H. Disturbed Excitation Energy Transfer in Arabidopsis Thaliana Mutants Lacking Minor Antenna Complexes of Photosystem II. *Biochimica et Biophysica Acta (BBA) - Bioenergetics* **2014**, *1837* (12), 1981–1988. https://doi.org/10.1016/j.bbabio.2014.09.011.

(3) Dall'Osto, L.; Cazzaniga, S.; Bressan, M.; Paleček, D.; Židek, K.; Niyogi, K. K.; Fleming, G. R.; Zigmantas, D.; Bassi, R. Two Mechanisms for Dissipation of Excess Light in Monomeric and Trimeric Light-Harvesting Complexes. *Nat Plants* **2017**, *3* (5), 17033. https://doi.org/10.1038/nplants.2017.33.

(4) Saccon, F.; Giovagnetti, V.; Shukla, M. K.; Ruban, A. V. Rapid Regulation of Photosynthetic Light Harvesting in the Absence of Minor Antenna and Reaction Centre Complexes. *J Exp Bot* **2020**, *71* (12), 3626–3637. https://doi.org/10.1093/jxb/eraa126.

(5) Belgio, E.; Johnson, M. P.; Jurić, S.; Ruban, A. V. Higher Plant Photosystem II Light-Harvesting Antenna, Not the Reaction Center, Determines the Excited-State Lifetime - Both the Maximum and the Nonphotochemically Quenched. *Biophys J* **2012**, *102* (12), 2761–2771. https://doi.org/10.1016/j.bpj.2012.05.004.

(6) Wilson, S.; Li, D.; Ruban, A. V. The Structural and Spectral Features of Light-Harvesting Complex II Proteoliposomes Mimic Those of Native Thylakoid Membranes. *J Phys Chem Lett* **2022**, *1*, 5683–5691. https://doi.org/10.1021/acs.jpclett.2c01019.

(7) Porra, R. J.; Thompson, W. A.; Kriedemann, P. E. Determination of Accurate Extinction Coefficients and Simultaneous Equations for Assaying Chlorophylls a and b Extracted with Four Different Solvents: Verification of the Concentration of Chlorophyll Standards by Atomic Absorption Spectroscopy. *Biochimica et Biophysica Acta (BBA) - Bioenergetics* **1989**, *975* (3), 384–394. https://doi.org/10.1016/S0005-2728(89)80347-0.

(8) Nishimura, M.; Akazawa, T. Photosynthetic Activities of Spinach Leaf Protoplasts. *Plant Physiol* **1975**, *55* (4), 712–716. https://doi.org/10.1104/pp.55.4.712.

(9) Brady, N. G.; Qian, S.; Nguyen, J.; O'Neill, H. M.; Bruce, B. D. Small Angle Neutron Scattering and Lipidomic Analysis of a Native, Trimeric PSI-SMALP from a Thermophilic Cyanobacteria. *Biochimica et Biophysica Acta (BBA) - Bioenergetics* **2022**, *1863* (7), 148596. https://doi.org/10.1016/j.bbabio.2022.148596.

(10) Korotych, O.; Mondal, J.; Gattás-Asfura, K. M.; Hendricks, J.; Bruce, B. D. Evaluation of Commercially Available Styrene-Co-Maleic Acid Polymers for the Extraction of Membrane Proteins from Spinach Chloroplast Thylakoids. *Eur Polym J* **2019**, *114* (June 2018), 485–500. https://doi.org/10.1016/j.eurpolymj.2018.10.035.

(11) Korotych, O. I.; Nguyen, T. T.; Reagan, B. C.; Burch-Smith, T. M.; Bruce, B. D. Poly(Styrene-Co-Maleic Acid)-Mediated Isolation of Supramolecular Membrane Protein Complexes from Plant Thylakoids. *Biochimica et Biophysica Acta (BBA) - Bioenergetics* **2021**, *1862* (3), 148347. https://doi.org/10.1016/j.bbabio.2020.148347.

(12) Cherepanov, D. A.; Brady, N. G.; Shelaev, I. V.; Nguyen, J.; Gostev, F. E.; Mamedov, M. D.; Nadtochenko, V. A.; Bruce, B. D. PSI-SMALP, a Detergent-Free Cyanobacterial Photosystem I, Reveals Faster Femtosecond Photochemistry. *Biophys J* **2020**, *118* (2), 337–351. https://doi.org/10.1016/j.bpj.2019.11.3391.

(13) Scheidelaar, S.; Koorengevel, M. C.; van Walree, C. A.; Dominguez, J. J.; Dörr, J. M.; Killian, J. A. Effect of Polymer Composition and PH on Membrane Solubilization by Styrene-Maleic Acid Copolymers. *Biophys J* **2016**, *111* (9), 1974–1986. https://doi.org/10.1016/j.bpj.2016.09.025.

(14) Ruban, A. V.; Lee, P. J.; Wentworth, M.; Young, A. J.; Horton, P. Determination of the Stoichiometry and Strength of Binding of Xanthophylls to the Photosystem II Light Harvesting Complexes. *Journal of Biological Chemistry* **1999**, *274* (15), 10458–10465. https://doi.org/10.1074/jbc.274.15.10458.

(15) Shukla, M. K.; Watanabe, A.; Wilson, S.; Giovagnetti, V.; Moustafa, E. I.; Minagawa, J.; Ruban, A. V. A Novel Method Produces Native Light-Harvesting Complex II Aggregates from the Photosynthetic Membrane Revealing Their Role in Nonphotochemical Quenching. *Journal of Biological Chemistry* **2020**, *295* (51), 17816–17826. https://doi.org/10.1074/jbc.RA120.016181.

(16) Wilson, S.; Johnson, M. P.; Ruban, A. V. Proton Motive Force in Plant Photosynthesis Dominated by ΔpH in Both Low and High Light. *Plant Physiol* **2021**, *187* (1), 263–275. https://doi.org/10.1093/plphys/kiab270.

(17) Schreiber, U.; Klughammer, C. New Accessory for the DUAL-PAM-100: The P515/535 Module and Examples of Its Application. *PAM Application Notes* **2008**, *10*, 1–10.

(18) Giovagnetti, V.; Ruban, A. V. The Mechanism of Regulation of the Photosystem I Cross-Section in the Pennate Diatom Phaeodactylum Tricornutum. *J Exp Bot* **2020**. https://doi.org/10.1093/jxb/eraa478.

(19) Johnson, M. P.; Ruban, A. V. Photoprotective Energy Dissipation in Higher Plants Involves Alteration of the Excited State Energy of the Emitting Chlorophyll(s) in the Light Harvesting Antenna II (LHCII). *Journal of Biological Chemistry* **2009**, *284* (35), 23592–23601. https://doi.org/10.1074/jbc.M109.013557.

(20) Fišerová, E.; Kubala, M. Mean Fluorescence Lifetime and Its Error. *J Lumin* **2012**, *132* (8), 2059–2064. https://doi.org/10.1016/j.jlumin.2012.03.038.

(21) Li, M.; Mukhopadhyay, R.; Svoboda, V.; Oung, H. M. O.; Mullendore, D. L.; Kirchhoff, H. Measuring the Dynamic Response of the Thylakoid Architecture in Plant Leaves by Electron Microscopy. *Plant Direct* **2020**, *4* (11), 1–14. https://doi.org/10.1002/pld3.280.

(22) Pfeiffer, S.; Krupinska, K. New Insights in Thylakoid Membrane Organization. *Plant Cell Physiol* **2005**, *46* (9), 1443–1451. https://doi.org/10.1093/pcp/pci156.

(23) Kirchhoff, H.; Hall, C.; Wood, M.; Herbstová, M.; Tsabari, O.; Nevo, R.; Charuvi, D.; Shimoni, E.; Reich, Z. Dynamic Control of Protein Diffusion within the Granal Thylakoid Lumen. *Proceedings of the National Academy of Sciences* **2011**, *108* (50), 20248–20253. https://doi.org/10.1073/pnas.1104141109.

(24) Schneider, C. A.; Rasband, W. S.; Eliceiri, K. W. NIH Image to ImageJ: 25 Years of Image Analysis. *Nat Methods* **2012**, *9* (7), 671–675. https://doi.org/10.1038/nmeth.2089.

(25) Belgio, E.; Ungerer, P.; Ruban, A. V. Light-Harvesting Superstructures of Green Plant Chloroplasts Lacking Photosystems. *Plant Cell Environ* **2015**, *38* (10), 2035–2047. https://doi.org/10.1111/pce.12528.

(26) Liu, Z.; Yan, H.; Wang, K.; Kuang, T.; Zhang, J.; Gui, L.; An, X.; Chang, W. Crystal Structure of Spinach Major Light-Harvesting Complex at 2.72 Å Resolution. *Nature* **2004**, *428* (6980), 287–292. https://doi.org/10.1038/nature02373.

(27) Wang, J.; Wolf, R. M.; Caldwell, J. W.; Kollman, P. A.; Case, D. A. Development and Testing of a General Amber Force Field. *J Comput Chem* **2004**, *25* (9), 1157–1174. https://doi.org/10.1002/jcc.20035.

(28) Prandi, I. G.; Viani, L.; Andreussi, O.; Mennucci, B. Combining Classical Molecular Dynamics and Quantum Mechanical Methods for the Description of Electronic Excitations: The Case of Carotenoids. *J Comput Chem* **2016**, *37* (11), 981–991. https://doi.org/10.1002/jcc.24286.

(29) Zhang, L.; Silva, D. A.; Yan, Y.; Huang, X. Force Field Development for Cofactors in the Photosystem II. *J Comput Chem* **2012**, *33* (25), 1969–1980. https://doi.org/10.1002/jcc.23016.

(30) Daskalakis, V.; Papadatos, S.; Kleinekathöfer, U. Fine Tuning of the Photosystem II Major Antenna Mobility within the Thylakoid Membrane of Higher Plants. *Biochimica et Biophysica Acta (BBA) - Biomembranes* **2019**, *1861* (12), 183059. https://doi.org/10.1016/j.bbamem.2019.183059.

(31) Daskalakis, V.; Papadatos, S.; Stergiannakos, T. The Conformational Phase Space of the Photoprotective Switch in the Major Light Harvesting Complex II. *Chemical Communications* **2020**, *56* (76), 11215–11218. https://doi.org/10.1039/d0cc04486e.

(32) Fan, M.; Li, M.; Liu, Z.; Cao, P.; Pan, X.; Zhang, H.; Zhao, X.; Zhang, J.; Chang, W. Crystal Structures of the PsbS Protein Essential for Photoprotection in Plants. *Nat Struct Mol Biol* **2015**, *22* (9), 729–735. https://doi.org/10.1038/nsmb.3068.

(33) Retegan, M.; Pantazis, D. A. Differences in the Active Site of Water Oxidation among Photosynthetic Organisms. *J Am Chem Soc* **2017**, *139* (41), 14340–14343. https://doi.org/10.1021/jacs.7b06351.

(34) Retegan, M.; Pantazis, D. A. Differences in the Active Site of Water Oxidation among Photosynthetic Organisms. *J Am Chem Soc* **2017**, *139* (41), 14340–14343.

(35) Mark, P.; Nilsson, L. Structure and Dynamics of the TIP3P, SPC, and SPC/E Water Models at 298 K. *Journal of Physical Chemistry A* **2001**, *105* (43), 9954–9960. https://doi.org/10.1021/jp003020w.

(36) Johnson, M. P.; Ruban, A. V. Restoration of Rapidly Reversible Photoprotective Energy Dissipation in the Absence of PsbS Protein by Enhanced ΔpH. *Journal of Biological Chemistry* **2011**, *286* (22), 19973–19981. https://doi.org/10.1074/jbc.M111.237255.

(37) Saccon, F.; Giovagnetti, V.; Shukla, M. K.; Ruban, A. V. Rapid Regulation of Photosynthetic Light Harvesting in the Absence of Minor Antenna and Reaction Centre Complexes. *J Exp Bot* **2020**, *71* (12), 3626–3637. https://doi.org/10.1093/jxb/eraa126.

(38) Noctor, G.; Rees, D.; Young, A.; Horton, P. The Relationship between Zeaxanthin, Energy-Dependent Quenching of Chlorophyll Fluorescence, and Trans-Thylakoid PH Gradient in

Isolated Chloroplasts. *Biochimica et Biophysica Acta (BBA) - Bioenergetics* **1991**, *1057* (3), 320–330. https://doi.org/10.1016/S0005-2728(05)80143-4.

(39) Rees, D.; Young, A.; Noctor, G.; Britton, G.; Horton, P. Enhancement of the ΔpH-Dependent Dissipation of Excitation Energy in Spinach Chloroplasts by Light-Activation: Correlation with the Synthesis of Zeaxanthin. *FEBS Lett* **1989**, *256* (1–2), 85–90. https://doi.org/10.1016/0014-5793(89)81723-5.

(40) Liu, C.; Rao, Y.; Zhang, L.; Yang, C. Identification of the Roles of Individual Amino Acid Residues of the Helix E of the Major Antenna of Photosystem II (LHCII) by Alanine Scanning Mutagenesis. *J Biochem* **2014**, mvu028.

(41) Townsend, A. J.; Saccon, F.; Giovagnetti, V.; Wilson, S.; Ungerer, P.; Ruban, A. V. The Causes of Altered Chlorophyll Fluorescence Quenching Induction in the Arabidopsis Mutant Lacking All Minor Antenna Complexes. *Biochimica et Biophysica Acta (BBA)-Bioenergetics* **2018**.

(42) Walters, R. G.; Ruban, A. V; Horton, P. Identification of Proton-Active Residues in a Higher Plant Light-Harvesting Complex. *Proceedings of the National Academy of Sciences* **1996**, *93* (24), 14204–14209.

(43) Liu, C.; Rao, Y.; Zhang, L.; Yang, C. Identification of the Roles of Individual Amino Acid Residues of the Helix E of the Major Antenna of Photosystem II (LHCII) by Alanine Scanning Mutagenesis. *J Biochem* **2014**, *156* (4), 203–210. https://doi.org/10.1093/jb/mvu028.

(44) Walters, R. G.; Ruban, A. V; Horton, P. Identification of Proton-Active Residues in a Higher Plant Light-Harvesting Complex. *Proc Natl Acad Sci U S A* **1996**, *93* (24), 14204–14209. https://doi.org/10.1073/pnas.93.24.14204.

(45) Townsend, A. J.; Saccon, F.; Giovagnetti, V.; Wilson, S.; Ungerer, P.; Ruban, A. V. The Causes of Altered Chlorophyll Fluorescence Quenching Induction in the Arabidopsis Mutant Lacking All Minor Antenna Complexes. *Biochimica et Biophysica Acta (BBA) - Bioenergetics* **2018**, *1859* (9), 666–675. https://doi.org/10.1016/j.bbabio.2018.03.005.

(46) Olsson, M. H. M.; SØndergaard, C. R.; Rostkowski, M.; Jensen, J. H. PROPKA3: Consistent Treatment of Internal and Surface Residues in Empirical p K a Predictions. *J Chem Theory Comput* **2011**, *7* (2), 525–537. https://doi.org/10.1021/ct100578z.

(47) Søndergaard, C. R.; Olsson, M. H. M.; Rostkowski, M.; Jensen, J. H. Improved Treatment of Ligands and Coupling Effects in Empirical Calculation and Rationalization of p K a Values. *J Chem Theory Comput* **2011**, *7* (7), 2284–2295. https://doi.org/10.1021/ct200133y.

(48) Daskalakis, V. Protein-Protein Interactions within Photosystem II under Photoprotection: The Synergy between CP29 Minor Antenna, Subunit S (PsbS) and Zeaxanthin at All-Atom Resolution. *Physical Chemistry Chemical Physics* **2018**. https://doi.org/10.1039/c8cp01226a.

(49) Liguori, N.; Campos, S. R. R.; Baptista, A. M.; Croce, R. Molecular Anatomy of Plant Photoprotective Switches: The Sensitivity of PsbS to the Environment, Residue by Residue. *J Phys Chem Lett* **2019**, *10* (8), 1737–1742. https://doi.org/10.1021/acs.jpclett.9b00437.

(50) Berendsen, H. J. C.; van der Spoel, D.; van Drunen, R. GROMACS: A Message-Passing Parallel Molecular Dynamics Implementation. *Comput Phys Commun* **1995**, *91* (1–3), 43–56. https://doi.org/10.1016/0010-4655(95)00042-E.

(51) Darden, T.; York, D.; Pedersen, L. Particle Mesh Ewald: An N ·log(N) Method for Ewald Sums in Large Systems. *J Chem Phys* **1993**, *98* (12), 10089–10092. https://doi.org/10.1063/1.464397.

(52) Hess, B.; Bekker, H.; Berendsen, H. J. C.; Fraaije, J. G. E. M. LINCS: A Linear Constraint Solver for Molecular Simulations. *J Comput Chem* **1997**, *18* (12), 1463–1472. https://doi.org/10.1002/(SICI)1096-987X(199709)18:12<1463::AID-JCC4>3.0.CO;2-H.

(53) Bussi, G.; Donadio, D.; Parrinello, M. Canonical Sampling through Velocity Rescaling. *Journal of Chemical Physics* **2007**, *126* (1). https://doi.org/10.1063/1.2408420.

(54) Parrinello, M.; Rahman, A. Polymorphic Transitions in Single Crystals: A New Molecular Dynamics Method. *J Appl Phys* **1981**, *52* (12), 7182–7190. https://doi.org/10.1063/1.328693.

(55) Wang, L.; Friesner, R. A.; Berne, B. J. Replica Exchange with Solute Scaling: A More Efficient Version of Replica Exchange with Solute Tempering (REST2). *Journal of Physical Chemistry B* **2011**, *115* (30), 9431–9438. https://doi.org/10.1021/jp204407d.

(56) Tribello, G. A.; Bonomi, M.; Branduardi, D.; Camilloni, C.; Bussi, G. PLUMED 2: New Feathers for an Old Bird. *Comput Phys Commun* **2014**, *185* (2), 604–613.

Wilson et al.
Supplementary Data Figure 1

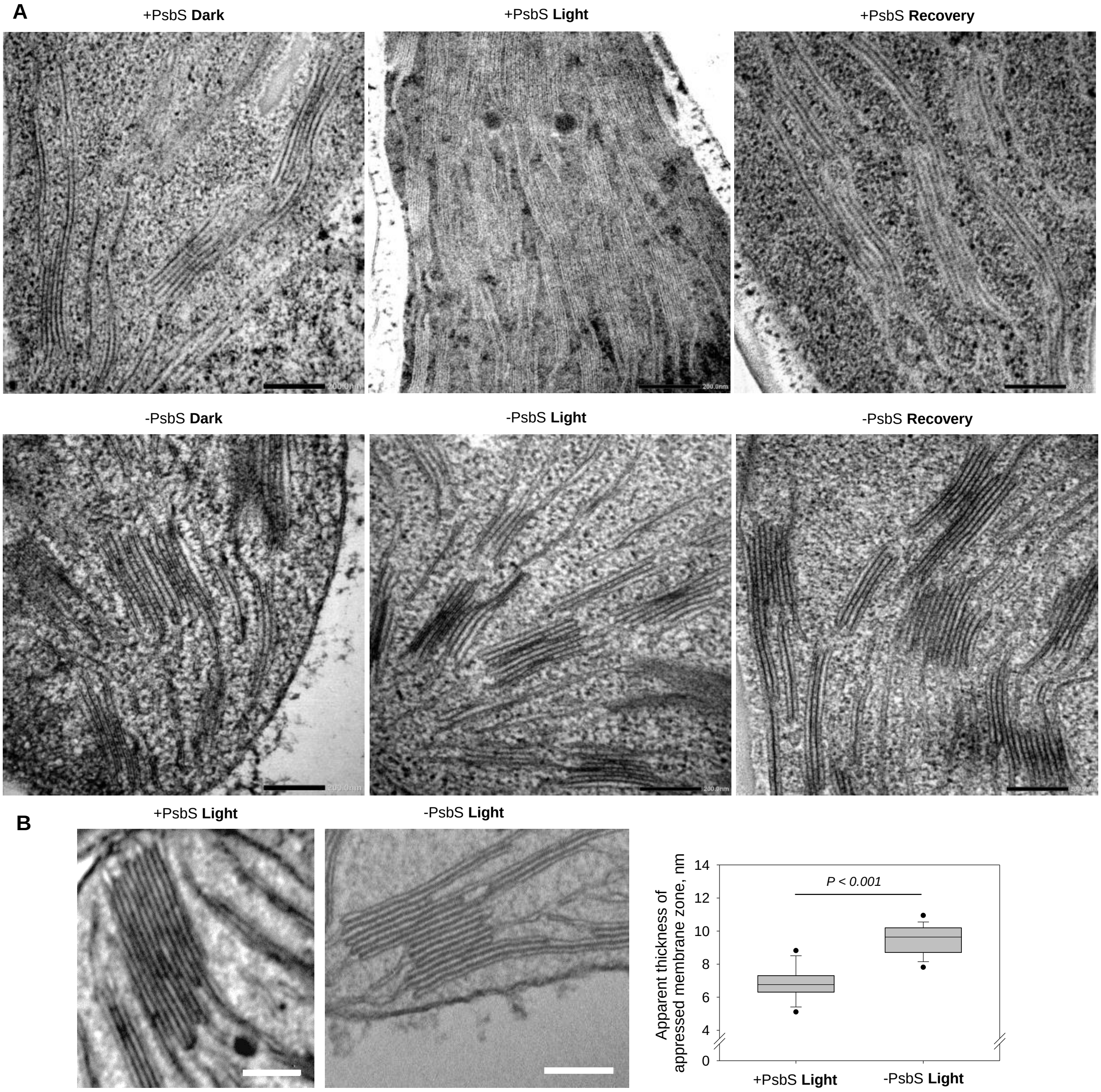

Supplementary Fig. 1 Cryo-immobilised and chemically-immobilised transmission electron micrographs of Arabidopsis leaves

(A) Micrographs of cryo-immobilised leaf sections. Micrographs taken at a magnification of x30,000 of thin-sections of +PsbS (top row) and –PsbS (bottom row) leaves in dark (left column), light (middle column), and recovery (right column) states. Black scale bar in the lower right of each micrograph was inserted from the microscope software and represents 200 nm.

(B) Micrographs of chemically-immobilised leaf sections. Micrographs taken at a magnification of x50,000 of thin-sections of +PsbS (left image) and –PsbS (right image) in the light state. Right box plot shows apparent thickness of appressed membrane zone (two bilayer plus the stromal gap). Statistical significance determined via Student's *t*-test (n = 38 – 41). White scale bar represents 100 nm.

Wilson et al.
Supplementary Data Figure 2

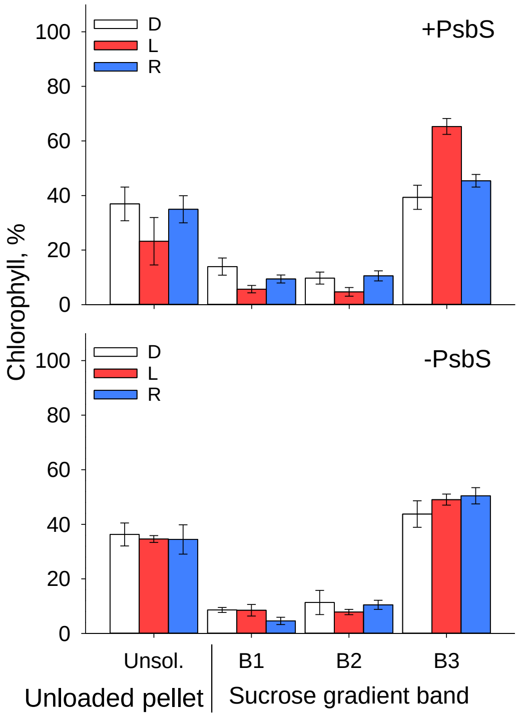

Supplementary Fig. 2 Chlorophyll distribution across sucrose gradients
Chlorophyll distribution of sucrose gradient bands and unloaded, unsolubilised material presented as a percentage of the total, calculated from absorption spectra. Data shown are the average of 3 – 4 independent repeats ± SEM. +PsbS condition shown (top panel) alongside –PsbS condition (bottom panel).

Wilson et al.
Supplementary Data Figure 3

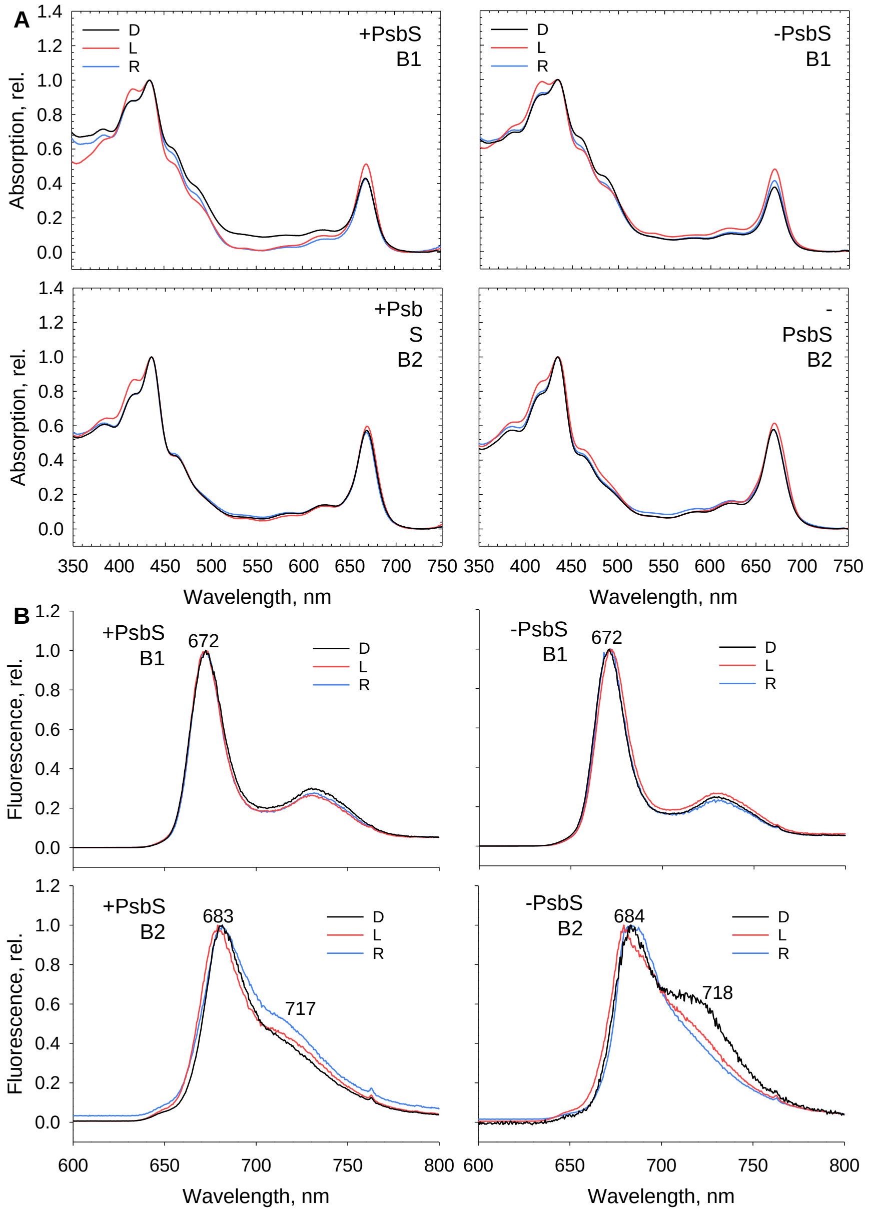

Supplementary Fig. 3 Spectroscopic analysis of B1 and B2 nanodisks

(A) Representative room-temperature absorption spectra of B1 and B2 bands from dark, light, and recovery-treated +PsbS (left column) and –PsbS (right column) conditions. Each spectrum is normalised to its maximum.

(B) Representative 77K fluorescence emission spectra of B1 and B2 bands from dark, light, and recovery-treated +PsbS (left column) and –PsbS (right column) conditions. Each spectrum is normalised to its maximum.

Wilson et al.
Supplementary Data Figure 4

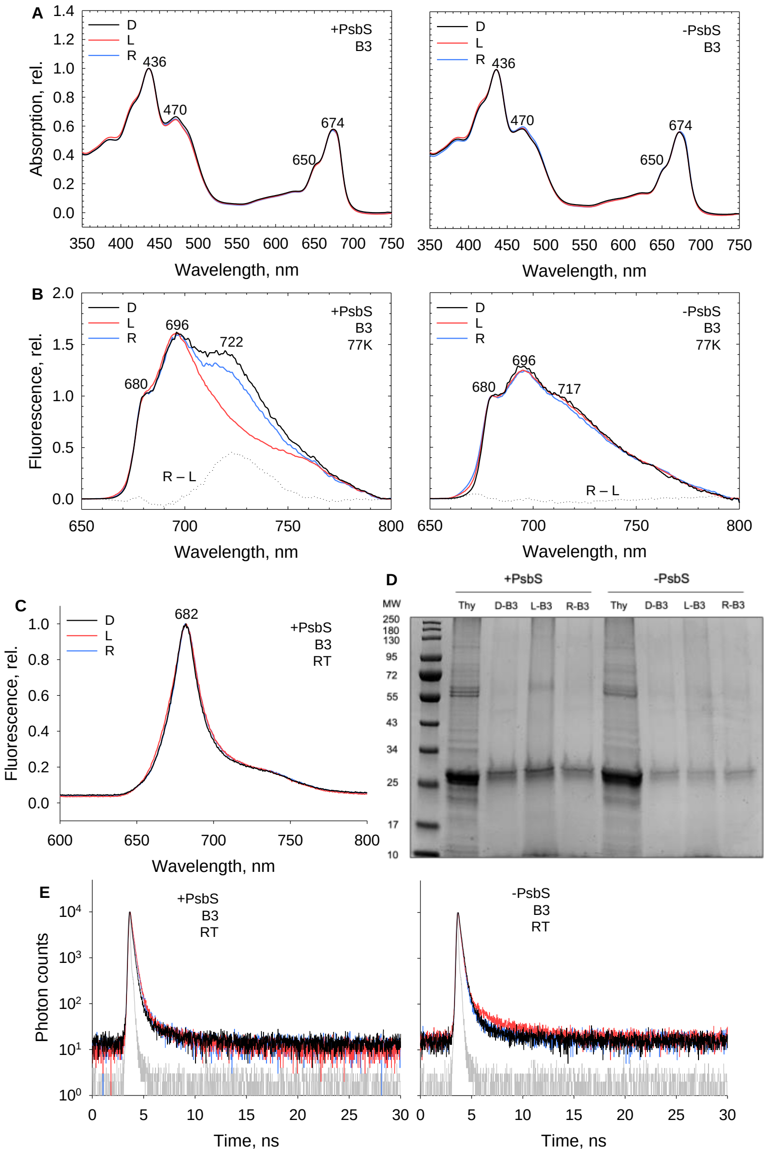

Supplementary Fig. 4 Spectroscopic and biochemical analysis of B3 nanodisks

(A) Representative room-temperature absorption spectra of the B3 band from dark, light, and recovery-treated +PsbS (left column) and –PsbS (right column) conditions. Each spectrum is normalised to its maximum.

(B) Representative 77K fluorescence emission spectra of the B3 band from dark, light, and recovery-treated +PsbS (left column) and –PsbS (right column) conditions. Each spectrum is normalised to its maximum.

(C) Representative room-temperature fluorescence spectra of the B3 band from dark, light, and recovery-treated +PsbS condition. Each spectrum is normalised to its maximum.

(D) 12% polyacrylamide SDS-PAGE gel run on thylakoid samples and B3 from each light treatment in +PsbS and –PsbS conditions. 1.5 µg total Chl loaded per lane of thylakoid sample, whilst 20 µL of the sucrose gradient band of interest was also loaded.

(E) Representative room-temperature fluorescence lifetimes measured through time-correlated single photon counting of the fluorescence emission detected at 680 nm, with the excitation at 468 nm. +PsbS condition shown in left panel, -PsbS condition shown in right panel. IRF shown in grey, dark in black, light in red, recovery in blue.

Wilson et al.
Supplementary Data Figure 5

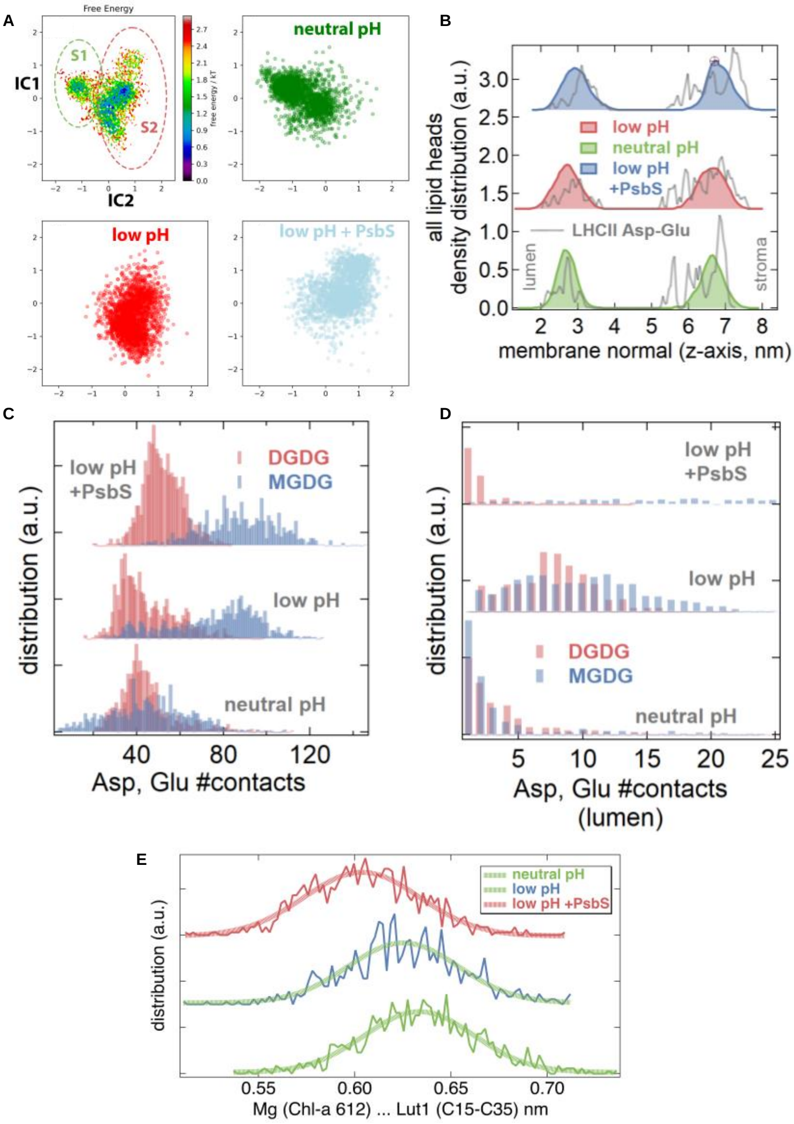

Supplementary Fig. 5 Extended molecular dynamics data

A) Weighted free energy surface of the LHCII along with the position of the sampled points in the different LHCII models on the same free energy space (tICA – Time-lagged Independent Component Analysis components IC1 and IC2). Energy values are in k_BT, with k_B being the Boltzmann constant and T being the temperature.

B) Lipid head density distributions along the membrane normal axis for neutral pH (lower section; green), low pH (middle section; red), and low pH + PsbS (upper section; blue), alongside LHCII Glu-Asp distributions for each condition (dashed grey line).

C) MGDG (blue) and DGDG (red) contacts with LHCII Glu-Asp residues for neutral pH (lower section), low pH (middle section), and low pH + PsbS (upper section).

D) Luminal MGDG (blue) and DGDG (red) contacts with LHCII Glu-Asp residues for neutral pH (lower section), low pH (middle section), and low pH + PsbS (upper section).

E) Interpigment distances in the LHCII terminal emitter locus Calculated as the distance of the central Mg atom of Chla 612 to the centre of the C15-C35 atoms of Lut1. Histograms for neutral pH (green; lower), low pH (blue; middle), and low pH +PsbS (red; upper) are shown for the locus close to the LHCII-PsbS cross section. Thus, in the presence of PsbS, this change in distance should trigger or facilitate a faster excitation energy transfer from the Chl-a Qy to the short-lived S1 or S* state of Lut-1, promoting the quenching.
